# Supplementary material for: ATP depletion in anthrax edema toxin pathogenesis
Source: PLoS Pathog. 2025 Apr 1;21(4):e1013017. doi: 10.1371/journal.ppat.1013017 (PMC11977985; doi:10.1371/journal.ppat.1013017)
Supplement: Attachment — Submitted filename: PLoS pathogens responses to reviewers.docx [file ppat.1013017.s004.docx]

Reviewers' comments:

**Reviewer #1**:

The manuscript "ATP depletion in anthrax edema toxin pathogenesis" by Liu et al. explores the mechanism underlying anthrax edema toxin mediated toxicity and host death. Pathogenic Bacillus anthracis produce two exotoxin virulence factors, called edema toxin (ET) and lethal toxin (LT), which are comprised of three proteins, edema factor (EF), lethal factor (LF) and protective antigen (PA). The respective cytosol acting factors (EF and LF) must combine with PA to make toxic complexes (ET and LT), since PA forms a membrane spanning channel that delivers the factors into the host cell's cytosol. While the traditional names of these toxins imply their biological activities (causing lethality and edema), it turns out both toxins can cause lethality, where ET is slightly more potent of a toxin than LT. The overt enzymatic activity of EF is an adenylate cyclase, converting ATP into cyclic AMP (cAMP); however, the precise mechanism whereby this activity causes lethality is poorly understood. The evidence presented indicates ATP depletion by EF is critical to toxin-mediated lethality rather than the downstream effects of increased cAMP concentration in host cells. This novel mechanism of ATP depletion for EF is exciting and may be general to the mode of action of other toxin adenylate cyclases produced by other pathogens.

Overall, this is an exciting finding and will inspire many future studies. I will summarize the key findings and then make my major critiques.

In earlier times isolated anthrax ET could not always be proven to be free of anthrax LT contamination. Thus any observed lethality of ET could have been attributed to contaminating LT. This fact changed when pure ET could be isolated. While this preparation causes the traditional swelling when injected subcutaneously, it also is a potent toxin that causes death and is even more potent than LT. The authors show here that highly pure ET causes death in mice in two days in an anthrax toxin receptor-dependent manner. Since internalized ET leads to cAMP production, they showed the metabolite accumulates in certain cells (excepting liver and kidney), and cAMP is also released by those cells into the extracellular medium. The authors next asked whether cAMP-dependent pathways were responsible for the observed toxicity. The looked at two cAMP targets, Epac and PKA. When these pathways were inhibited ET toxicity remained, thus indicating cAMP itself was not responsible for observed toxicity. The next insight made was the observation that ET mediated cell rounding only occurred in an anthrax toxin receptor-dependent manner. The cell rounding phenotype correlated with a depletion of cellular ATP pool by the fact that EF is an extremely efficient adenylate cyclase rapidly converting ATP to cAMP. Finally, ET is a more potent adenylate cyclase than the indirect activation of that pathway achieved by cholera toxin likely because cholera toxin relies on endogenous adenylate cyclase activity.

Thank you so much for your very positive view on our manuscript!

Major points:

Unlike LT (which has observed cardiovascular toxicity leading to host death), is the toxicity of ET attributable to other organ failure outside of the heart, since according to the author's initial observation cAMP does not accumulate in the heart and presumably ATP is not as depleted there as in other tissues? Make a little more effort to explain the potential pathways altered by ATP depletion which would lead to host death. While the companion LT study showing the toxic effects are likely metabolic dysfunction of the cardiovascular system has not been published, maybe still make some effort to explain why ET is needed if LT is already capable of causing death on its own due to also its ability to also interfere with central metabolism and energy status of the cell.

Our response:

We added brief discussion for this issue in the Discussion section (2^nd^ to the last paragraph) in the revised manuscript.

Minor points:

Page 4, the sentence, "Due to the similarity of ET and cholera toxin in their ability to cause fluid loss, it was believed Hthe two toxins had similar mechanisms of action", has a typo at "Hthe".

Our response:

Thank you. We corrected this error in the revised manuscript.

Page 9, the sentence, "Since anthrax ET is an adenylate cyclase, can also cause intestinal fluid accumulation and tissue edema (21, 24), it has been postulated that the cAMP-PKA-CFTR pathway may also mediate the in vivo toxicity of ET (4, 5)", remove the comma and inert "and" before "can" and after "cyclase".

Our response:

Thank you. We have modified this sentence accordingly in the revised manuscript.

Page 21, Figure S1A. "Vehcile" in figure legend is spelled wrong.

Our response:

Thank you. We corrected this error in the revised manuscript.

Figure 5 and Figure S2A. Point size is rather small on legends and axes labels.

Our response:

Thank you for pointing out this. We have increased the font sizes in these figures in the revised manuscript. (now Fig. 5 and Fig. S3)

**Reviewer #2**:

This manuscript provides a fresh perspective on the mechanism of toxicity associated with anthrax edema toxin (ET). ET is an adenylate cyclase which converts ATP to cAMP. It is well established that cholera toxin increases cAMP and that this increase leads to the activation of protein kinase A (PKA), which in turn activates the cystic fibrosis conductance regulator (CFTR), which in turn leads to chloride efflux, water loss, and diarrhea. It has been shown that ET-associated cAMP increases can also activate PKA and therefore it has been assumed that the toxicity of ET is due to the negative consequences of unregulated CFTR activation.

In this manuscript, the authors note that while ET is a highly efficient adenylate cyclase (AC), capable of converting 1000-2000 molecules of ATP to cAMP per second, CT action depends on the availability of endogenous AC’s, and therefore the rate of ATP conversion to cAMP is limited by AC availability. They propose and provide evidence to suggest that while ET is capable of PKA-dependent CFTR activation, the in vivo toxicity of ET is a result of the rapid ATP depletion. This is a significant and conceptionally innovative advance for the field. However, there are two key points that need to be addressed.

Thank you so much for your positive view on our manuscript! We have carefully addressed your concerns as described below.

1. The first is experimental. The key experiment in Figure 2 lacks a critical control (described below).

Our response:

Thank you for pointing out this. This is a common concern also pointed by the 3^rd^ reviewer. We have carefully addressed this concern by doing additional experiments as described below.

2. Provided this is addressed, the second key point is that this paper is describing intoxication, not infection. The authors do not present any evidence that either ET mechanism (CFTR activation and/or ATP depletion) contributes to the pathogenesis of B. anthracis in vivo. Instead, they are intoxicating mice with very high doses (20 ug) of purified ET proteins. Given the regulatory challenges associated with select agent work, I would not expect the authors to provide experimental evidence in the context of an infection model. However, a revision should clearly indicate that the results should be interpreted in the context of intoxication, not infection. If there are robust data from the literature that link the intoxication data to infection models, that literature should be described as it would strengthen the manuscript.

Our response:

Thank you for your understanding of the many limitations regarding using select agents. We have carefully clarified through textual modification that the current manuscript described a work using an reductionist approach for understanding the pathogenesis of anthrax ET, which has been much less studied compared to anthrax lethal toxin, and the results need to be further confirmed in studies using virulent *B. anthracis* strains.

Major points:

1. The experiments described in Figure 2B- 2G do not have a control to establish that the drugs were present and acting as expected in the in vivo context. While there are probably multiple ways to address this, one compelling experiment would be to perform the same experiment with cholera toxin. According to the model, one would expect to see the drugs have an impact on cholera toxin induced edema. Similarly, testing the effect of CT in the experiment shown in 2H or 2I would provide evidence that the knock-out animal is functioning as anticipated.

Our response:

Thank you for this valid concern. We performed additional experiments, showing that the PKA inhibitor H89 can work efficiently in vivo in diminishing ET-induced activation of PKA-CREB pathway in mice (new Fig. 2C).

Since CFTR-/- mice are extremely hard to breed, our lab discontinue these mice to save research resource. Instead, since both CT and ET cause tissue edema, we performed side-by-side footpad edema experiments using ET and CT, along with PAK inhibitor H89 and CFTR inhibitor 172. The results of these new experiments appear to show the more pronounced efficacies of these inhibitors in diminishing edema caused by these toxins, in particular CT. The new data were included in Figs. S2, A-D in the revised manuscript.

Minor points:

Line 3 on first page of RESULTS: systematically should be systemically

Our response:

Thank you. We corrected this error in the revised manuscript.

**Reviewer #3**:

The manuscript by Liu et al. presents data on the effects of Bacillus anthracis edema toxin (ET) on anthrax pathogenesis and tissue edema formation. While the data are original, they are not entirely convincing and the manuscript has holes. Although the authors observed ATP depletion and cell death induced by ET in vitro—suggesting effects beyond the conventional cAMP-dependent toxin mechanisms—they did not demonstrate these findings in a relevant animal model. Additionally, the comparison between ET and cholera toxin (CT) is unclear throughout the text. Overall, the results seem to negate hypotheses more than offering a cohesive set of data supporting the role of ATP depletion in anthrax pathogenesis.

Thank you for your constructive comments, which we have addressed carefully as shown below. We hope you find our revisions are responsive.

Major points:

- On most panels of the figure 2 there is a lack of a positive control showing that H89 and CFTRinh172 are effective at the right concentration in the model to counteract a toxin. The authors cannot tell if the result is negative due to an inefficient model or a lack of effect of the inhibitor on the effector of the toxin.

Our response:

This is a valid and a common concern also raised by the 2^nd^ expert reviewer. Please see our above response to the major point #1 of the 2^nd^ reviewer.

- On figure 5 why is there such a lethality with ET and not with LT? How the authors reconcile the absence of lethal effect of LT. MTT test is rather a rough method. It would have been more interesting to address the cause of cell death (apoptosis, necrosis, pyroptosis, efferocytosis, etc...)

Our response:

Studies on anthrax toxins have long been focused on LT rather than ET. This was partially due to the much earlier availability of highly purified LF protein vs. EF protein.

Upon high quality of EF protein becoming available to the research community, ET was found to be lethal to mice (even more lethal than the same amount of LT), causing extensive tissue lesions and rapid lethality in mice. The ET-induced lethality was confirmed by multiple studies (REFs 26-29, 49).

The prevailing view in anthrax field is that:

Although LT is lethal to mice, the toxin often does not directly kill many cell types. LF targets MEKs 1 to 4, and 6, thus inhibiting the three MAPK pathways, i.e., the ERK, p38, and JNK pathways. Although LT is inhibitory on proliferation and growth of many cell types, those cells could survive through other parallel signaling pathways (mTOR). Thus, only a few cells thus are very sensitive to LT, including human cancer cells with the BRAF^V600E^ mutation (thereby are addictive to ERK pathway for survival) and macrophages from certain rat and mouse strains containing LT-sensitive NLRP1 proteins (can be rapidly killed by LT through pyroptosis).

We performed flow cytometry following PI/anexin V-staining, and found that ET but not LT could induce apoptosis of NIH3T3 cells (new Fig. S3).

- Finally, the manuscript lacks a demonstration that ATP depletion is the cause of pathogenesis. While the authors observe cell death in vitro, no evidence is provided for this effect in vivo. Moreover, edema toxin (ET) is not known to induce necrosis in animal models (unlike lethal toxin, LT), and I would be surprised if ET's effects were mediated by cell death

Our response:

The prevailing view in anthrax field is that ET is indeed lethal, causing tissue necrosis in mice (REFs 26-29, 49). Please see our above augment and the literature cited.
